# Supplementary material for: Indole Alkaloids and Chromones from the Stem Bark of Cassia alata and Their Antiviral Activities
Source: Molecules. 2022 May 13;27(10):3129. doi: 10.3390/molecules27103129 (PMC9144915; doi:10.3390/molecules27103129)
Supplement: Supplementary file 1 [file molecules-27-03129-s001.zip › molecules-1697523-supplementary.pdf]

Indole Alkaloids and Chromones from The Stem Bark of *Cassia alata* and Their Antiviral Activities

Pei-Song Yang[1,2], Jia-Meng Dai[1,2], Xue-Jiao Gu[1], Wen Xiong[2], De-Quan Huang[1], Shi-Yu Qiu[1], Jun-Na Zheng[1], Yong Li[2], Feng-Xian Yang[1,2,*], Min Zhou[1,*]

[a] *Key Laboratory of Chemistry in Ethnic Medicinal Resources, State Ethnic Affairs Commission & Ministry of Education, Yunnan Minzu University, Kunming 650031, P.R. China. E-mail: jszxtg_2015@163.com, zhouminynun@163.com;*

[b] *Yunnan Key Laboratory of Tobacco Chemistry, China tobacco yunnan industrial Co., Ltd, Kunming 650231, P.R. China. E-mail: 2091085534@qq.com.*

[*]Correspondence: jszxtg_2015@163.com (M. Z.); zhouminynun@163.com (M. Z.); yangfengxian90@163.com (F.-X. Y.);

Tel.: +86-0871-65919542 (M. Z.); Fax: +0871-65919542 (M. Z.).

Contents

Figure S1. ^{13}C and DEPT NMR spectrum of alataindolein A (**1**)

Figure S2. ^{1}H NMR spectrum of alataindolein A (**1**)

Figure S3. HSQC NMR spectrum of alataindolein A (**1**)

Figure S4. HMBC NMR spectrum of alataindolein A (**1**)

Figure S5. ^{13}C and DEPT NMR spectrum of alataindolein B (**2**)

Figure S6. ^{1}H NMR spectrum of alataindolein B (**2**)

Figure S7. ^{13}C and DEPT NMR spectrum of alataindolein C (**3**)

Figure S8. ^{1}H NMR spectrum of alataindolein C (**3**)

Figure S9. ^{13}C and DEPT spectrum of alatachromone A (**4**)

Figure S10. ^{1}H NMR spectrum of alatachromone A (**4**)

Figure S11.HSQC spectrum of alatachromone A (**4**)

Figure S12. HMBC spectrum of alatachromone A (**4**)

Figure S13. ^{13}C and DEPT NMR spectrum of alataindolein D (**5**)

Figure S14. ^{1}H NMR spectrum of alataindolein D (**5**)

Figure S15. HSQC NMR spectrum of alataindolein D (**5**)

Figure S16. HMBC NMR spectrum of alataindolein D (**5**)

Figure S17. The antiviral inhibition rates tested by half-leaf method for compounds **2**–**4**

Figure S18. The protective effects of compounds **2**–**4** on TMV

Figure S1. ^{13}C and DEPT NMR spectrum of alataindolein A (**1**)

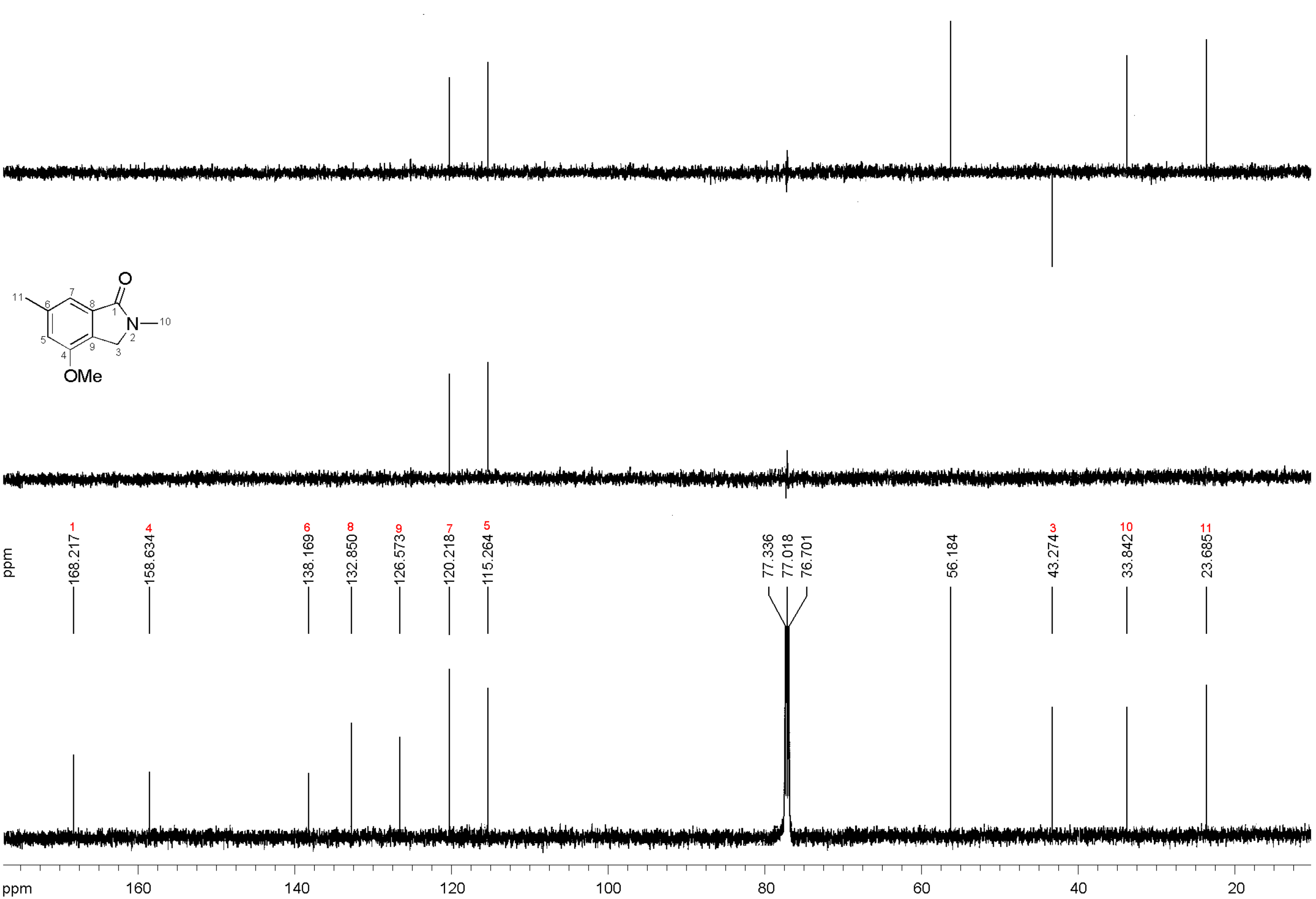

Figure S2. ^{1}H NMR spectrum of alataindolein A (**1**)

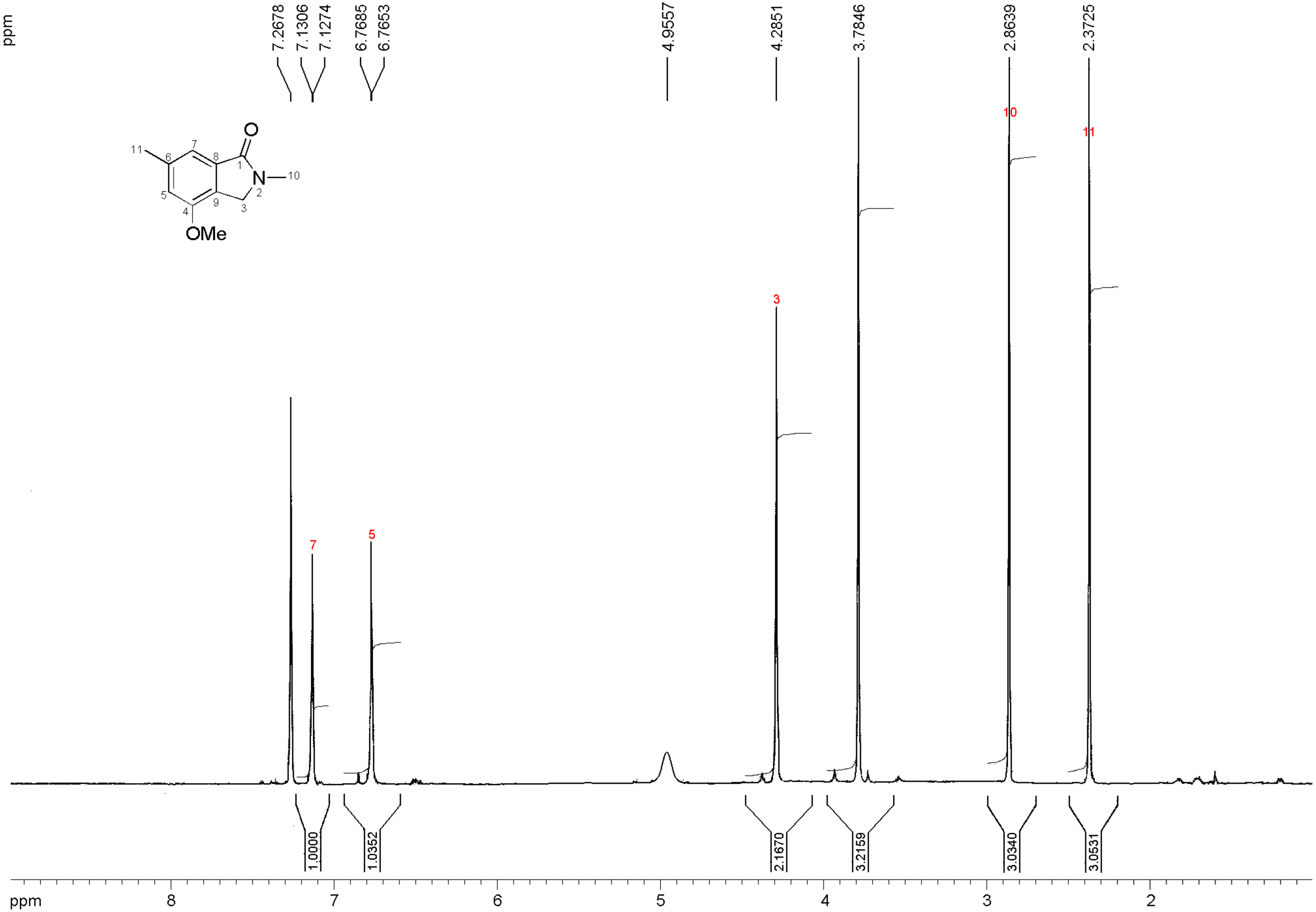

Figure S3. HSQC NMR spectrum of alataindolein (**1**)

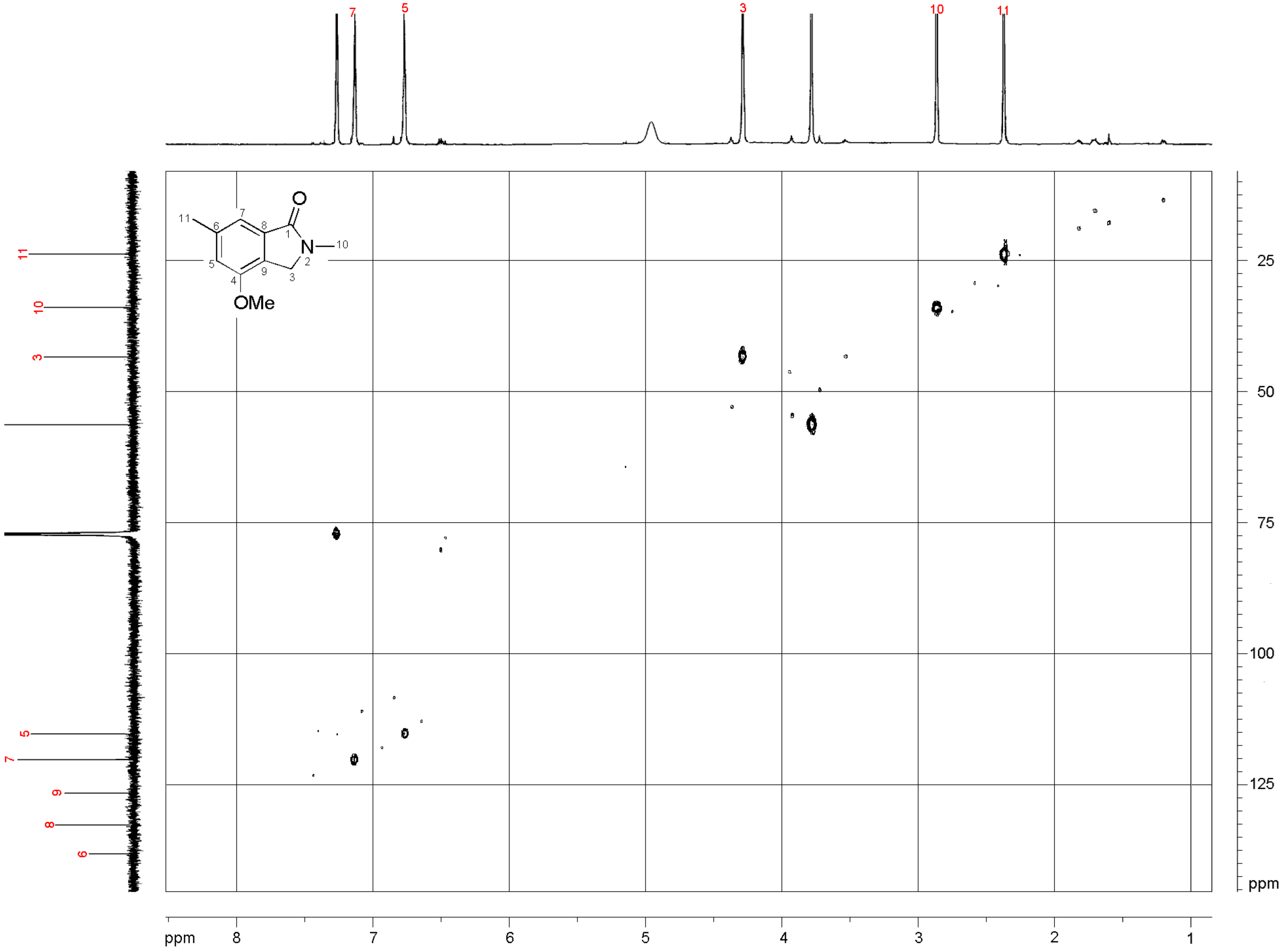

Figure S4. HMBC NMR spectrum of alataindolein A (**1**)

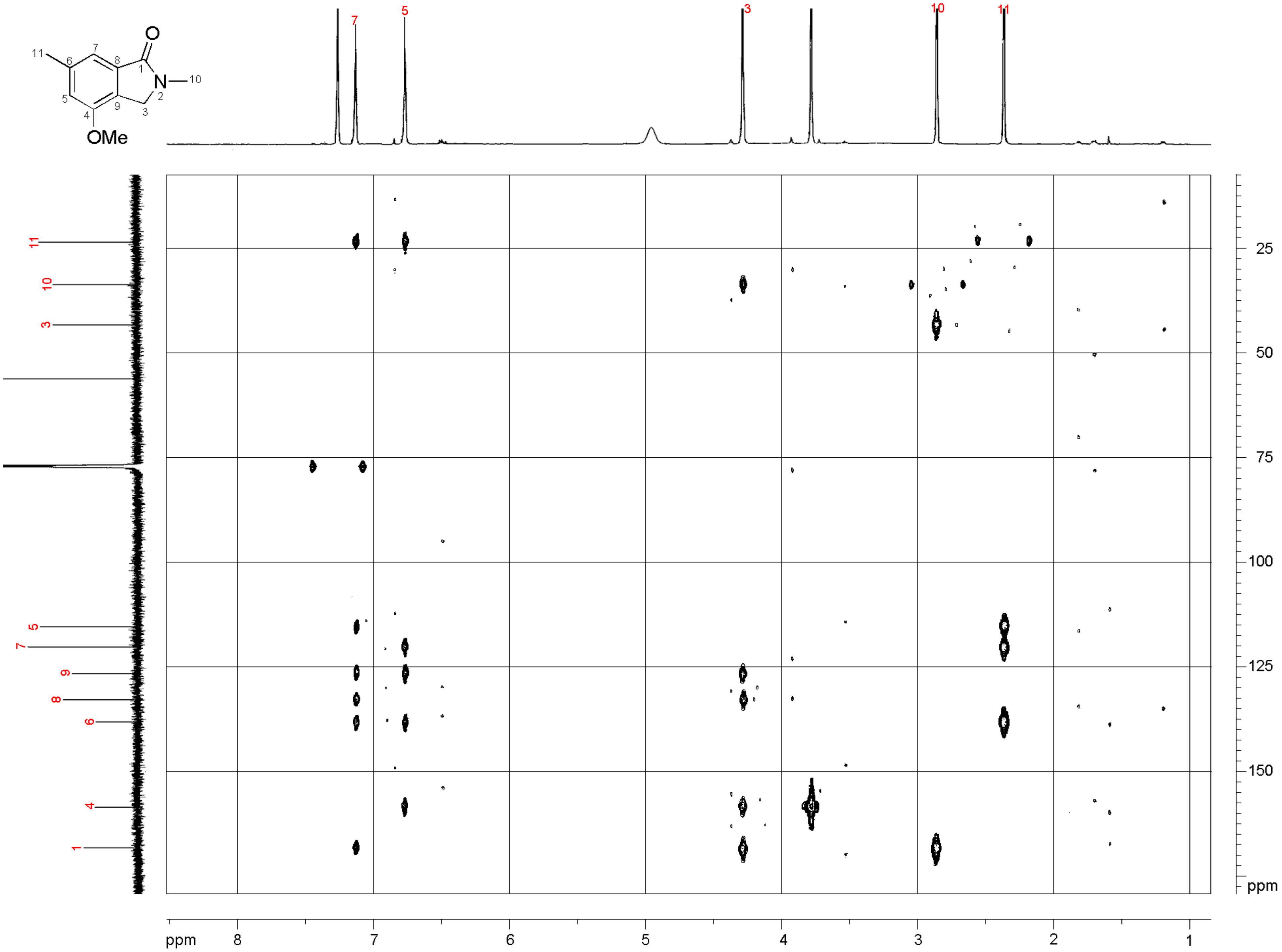

Figure S5. ^{13}C and DEPT NMR spectrum of alataindolein B (**2**)

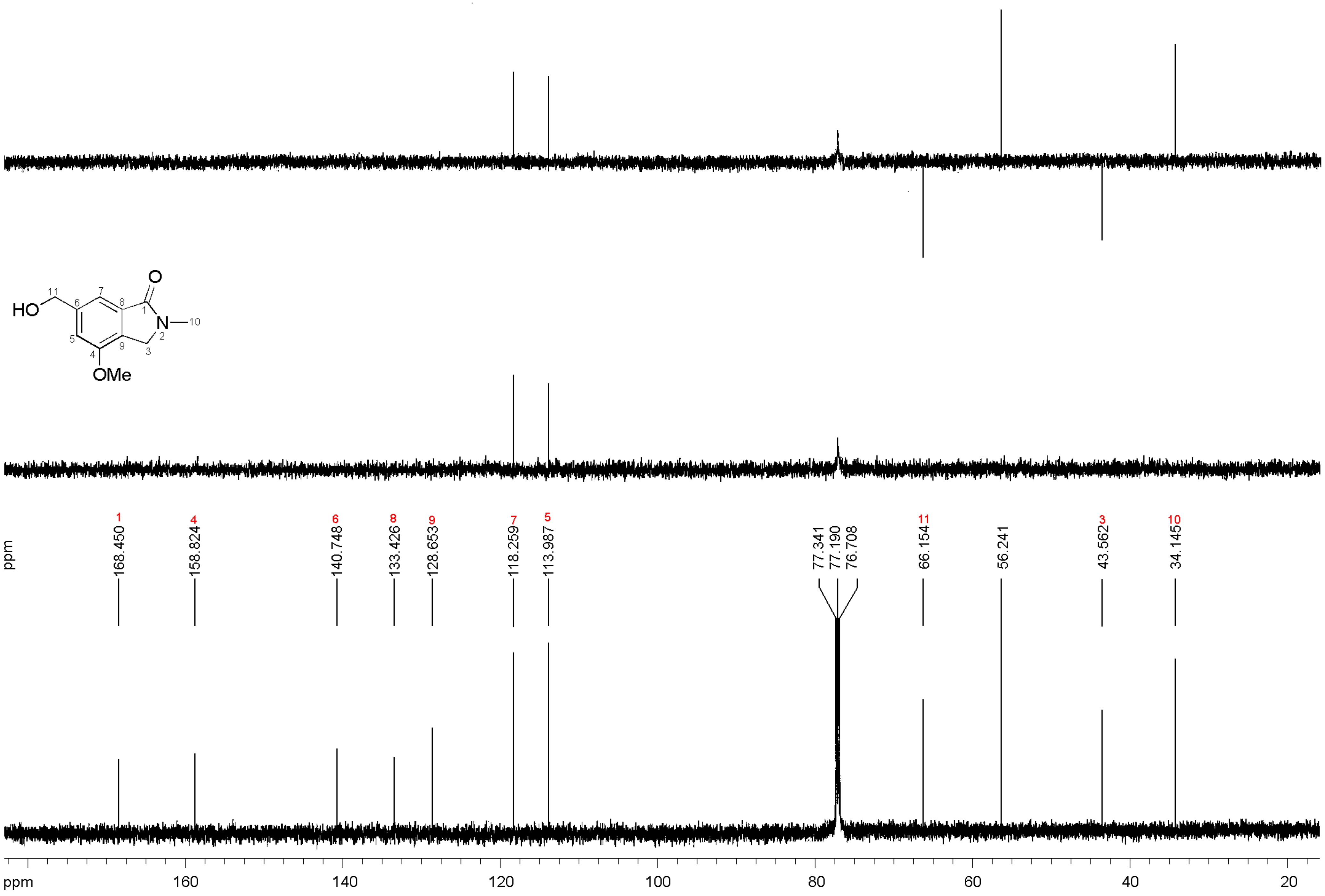

Figure S6. ^{1}H NMR spectrum of alataindolein B (**2**)

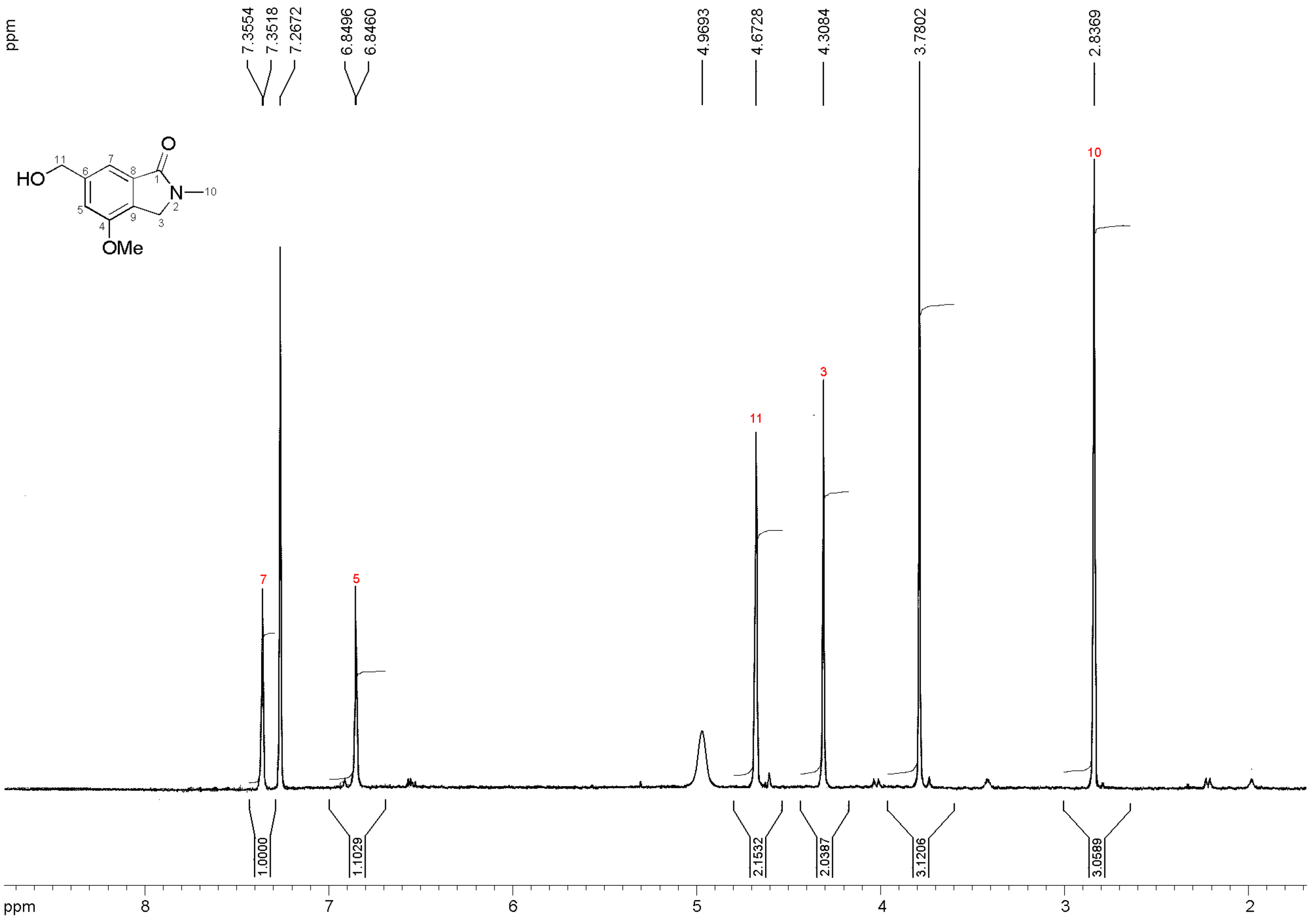

Figure S7. ^{13}C and DEPT NMR spectrum of alataindolein C (**3**)

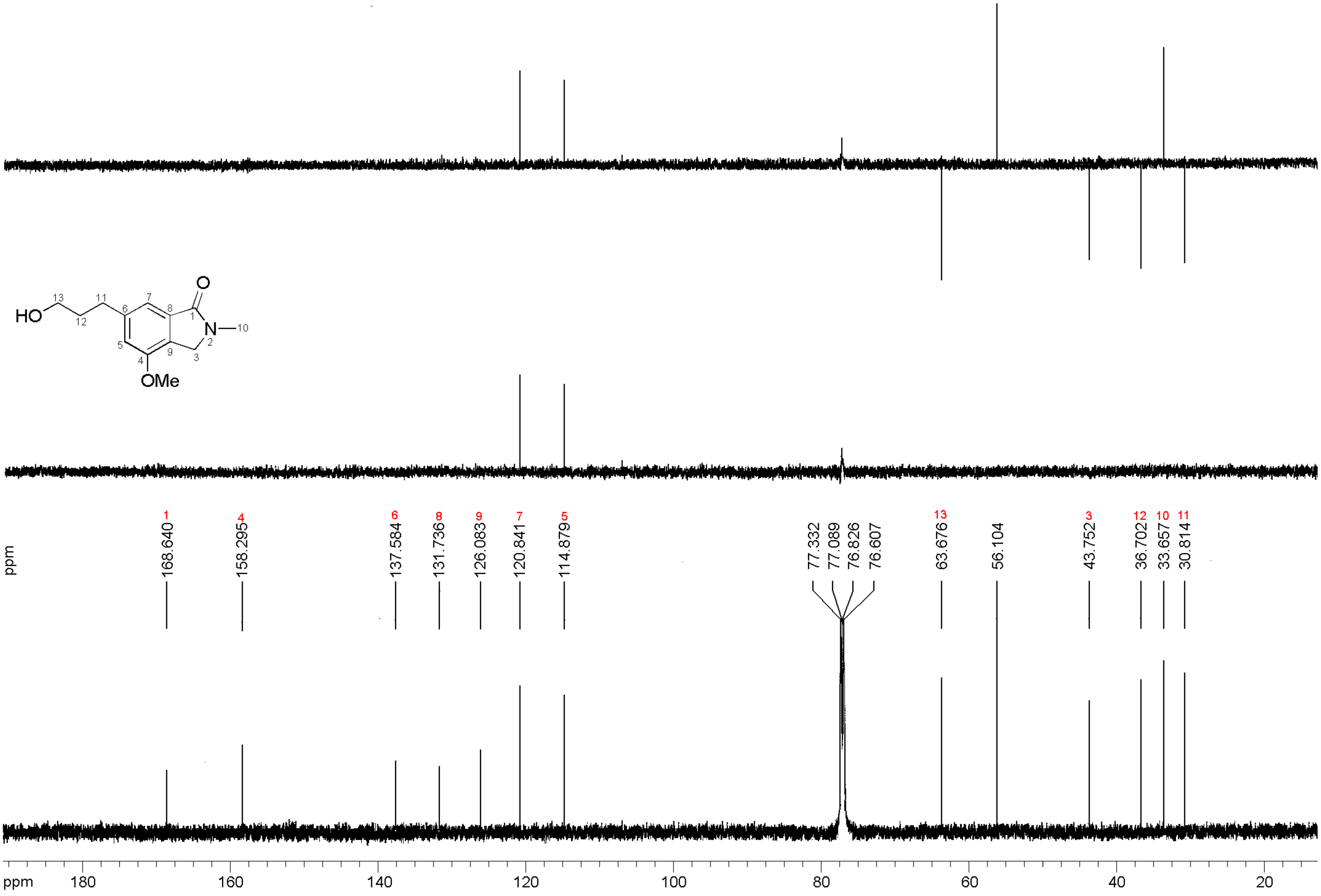

Figure S8. ^{1}H NMR spectrum of alataindolein C (**3**)

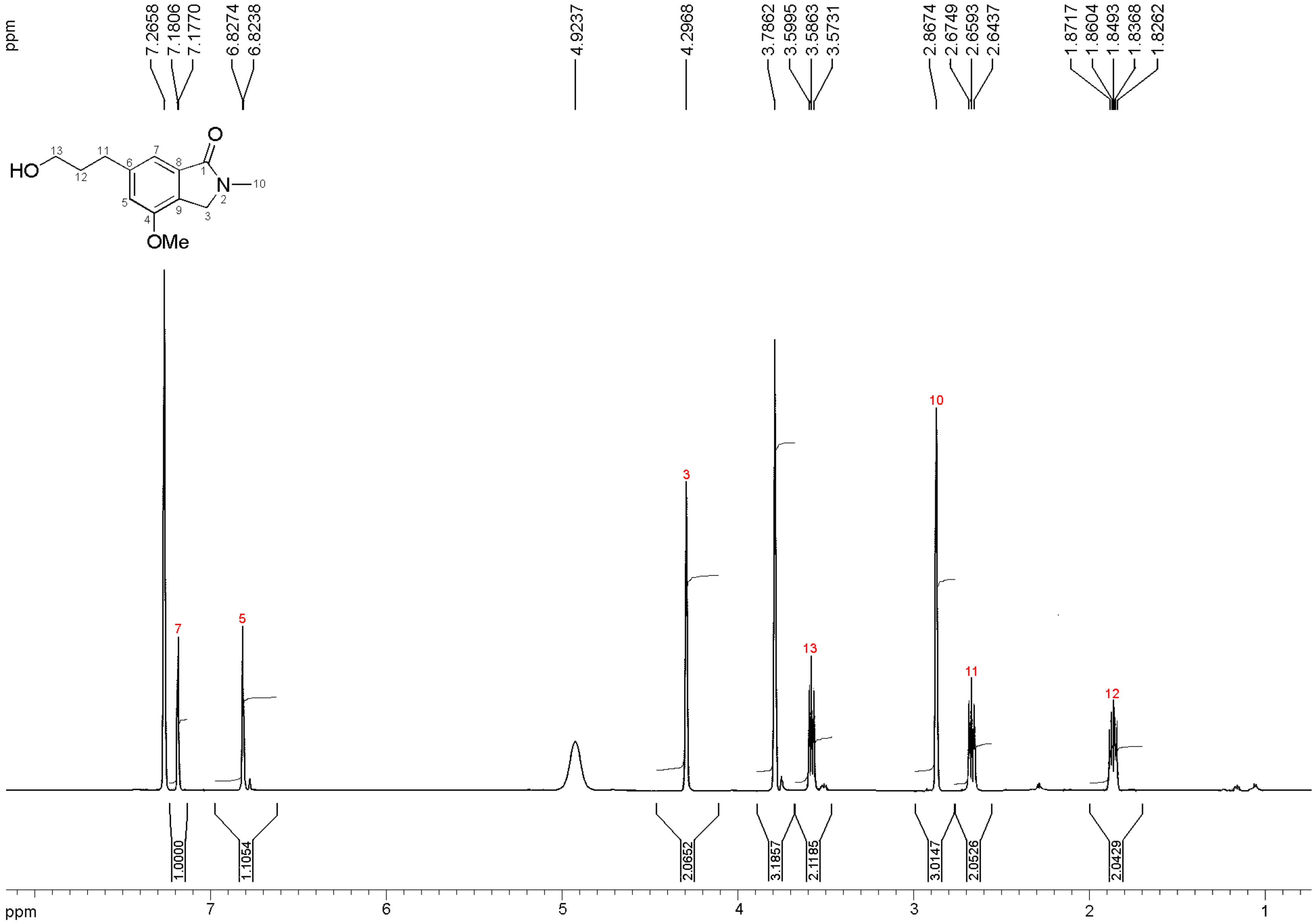

Figure S9. ^{13}C and DEPT spectrum of alatachromone A (**4**)

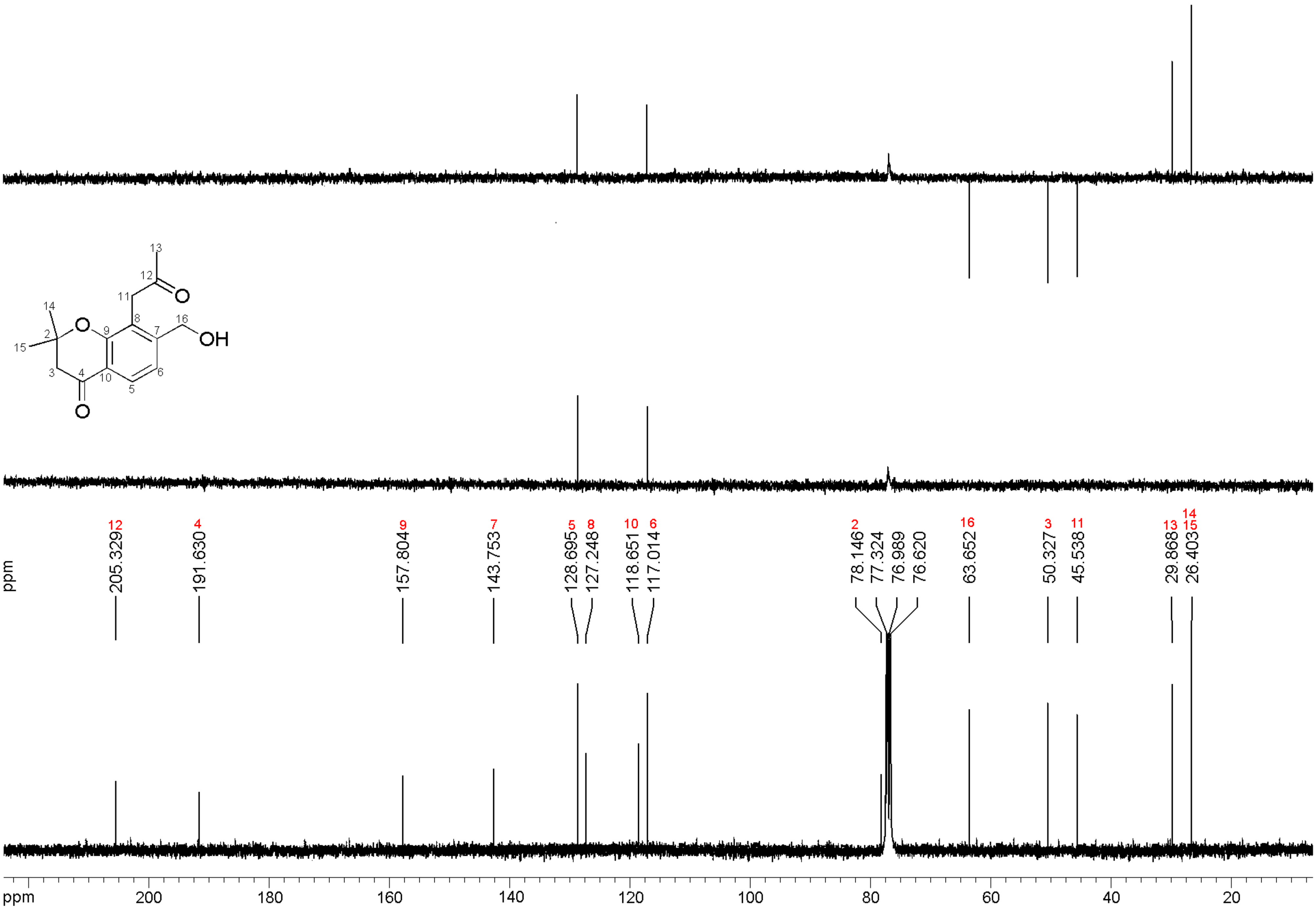

Figure S10. ^{1}H NMR spectrum of alatachromone A (**4**)

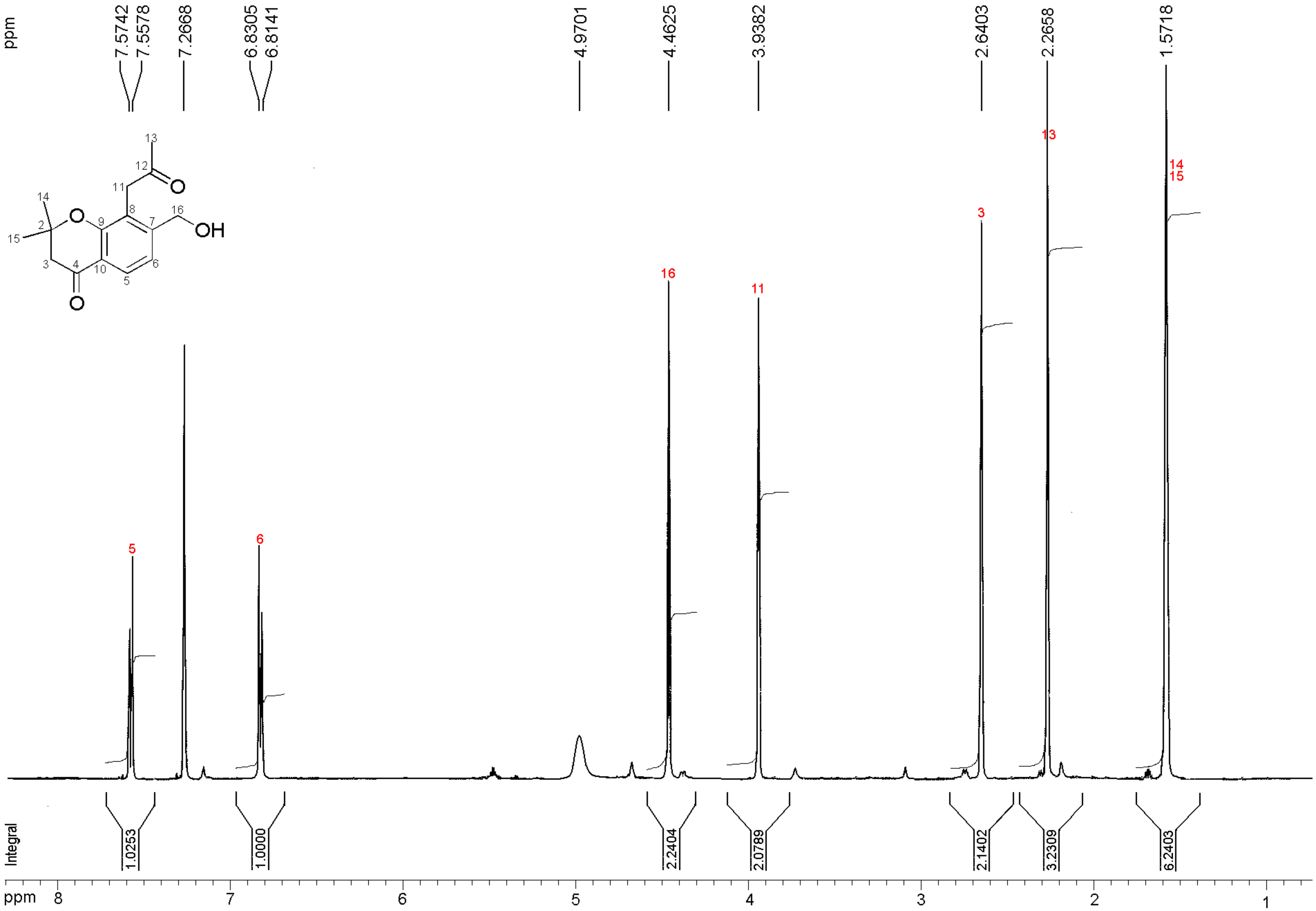

Figure S11. HSQC spectrum of alatachromone A (**4**)

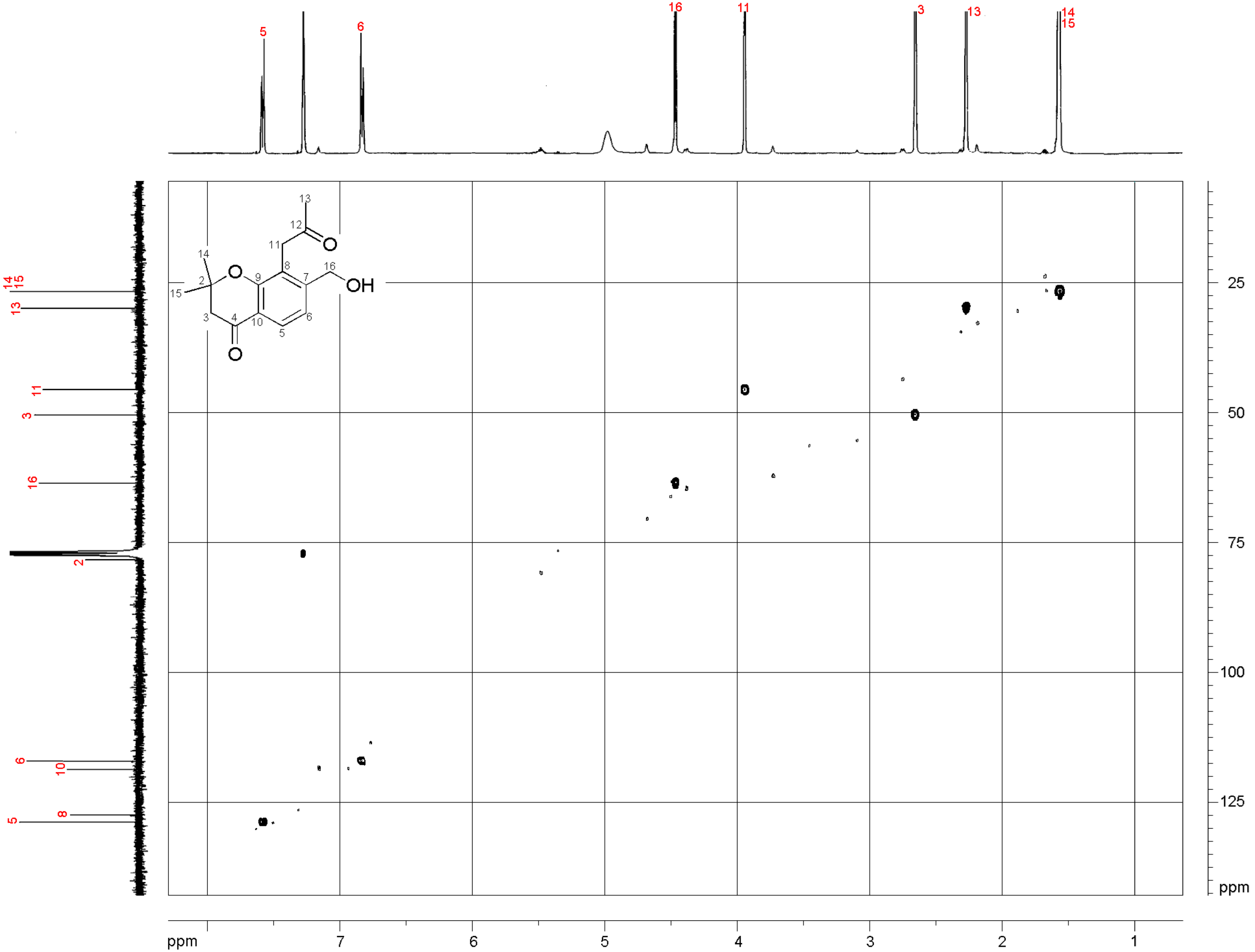

Figure S12. HMBC spectrum of alatachromone A (**4**)

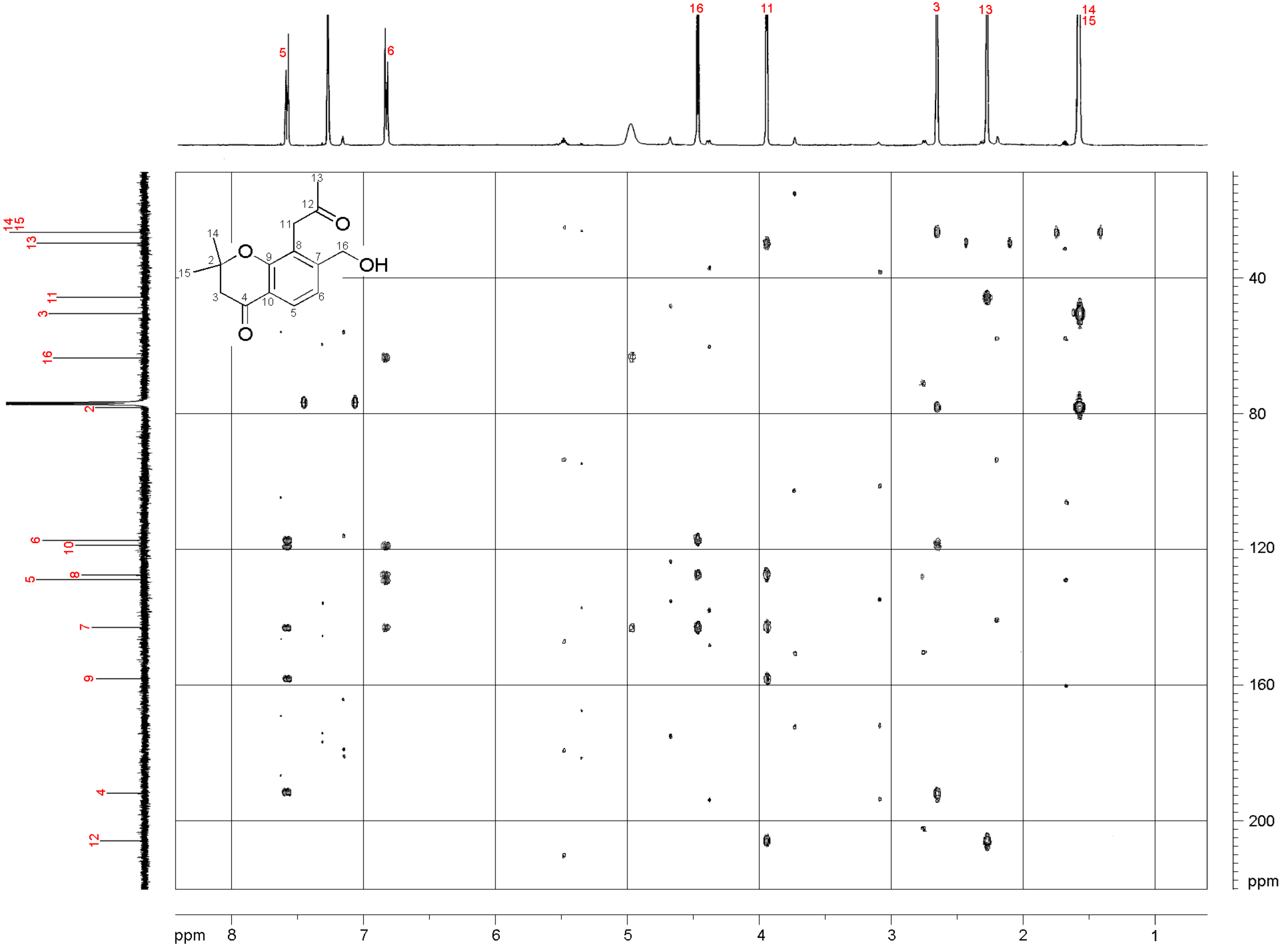

Figure S13. ^{13}C and DEPT NMR spectrum of alataindolein D (**5**)

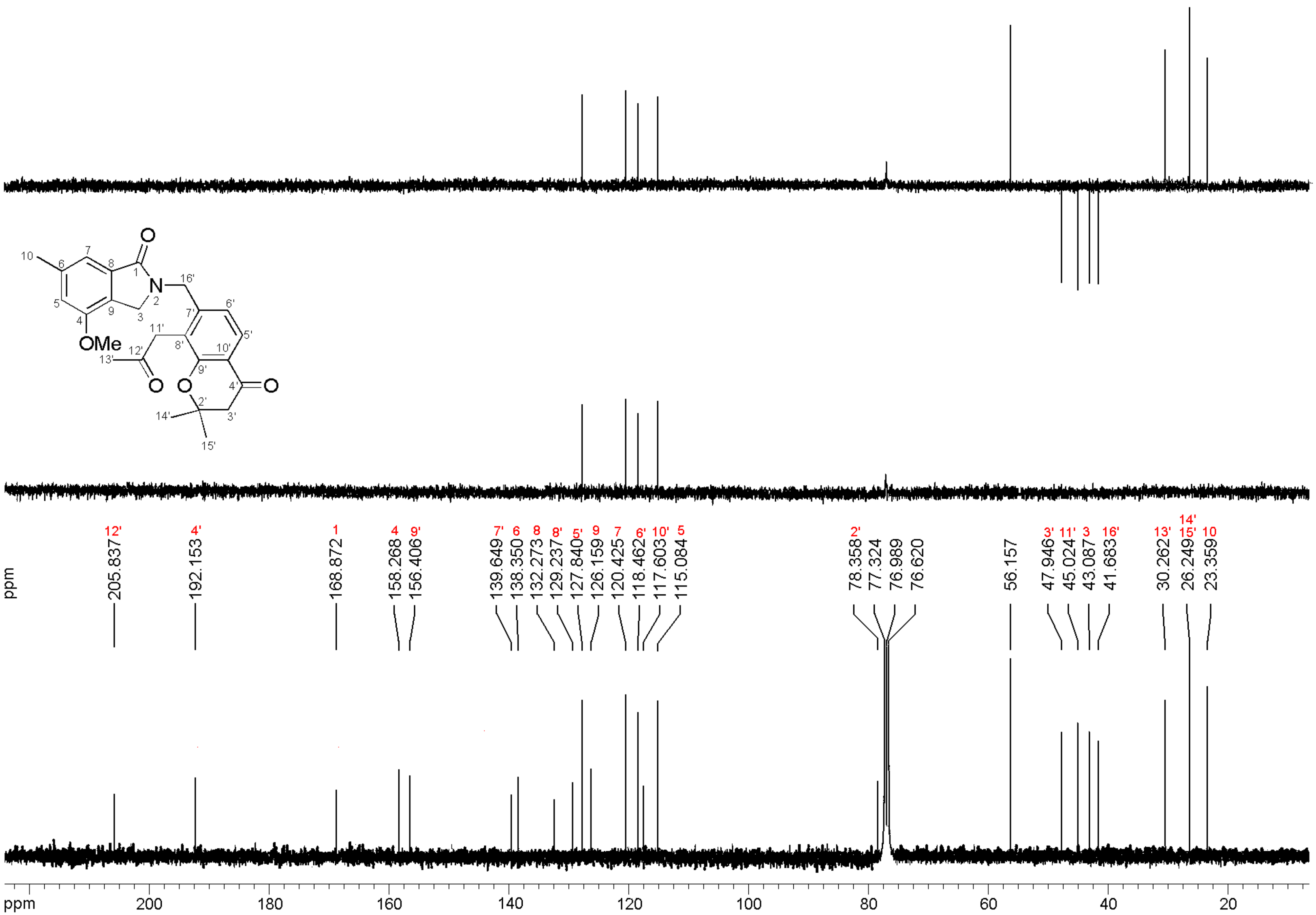

Figure S14. ^{1}H NMR spectrum of alataindolein D (**5**)

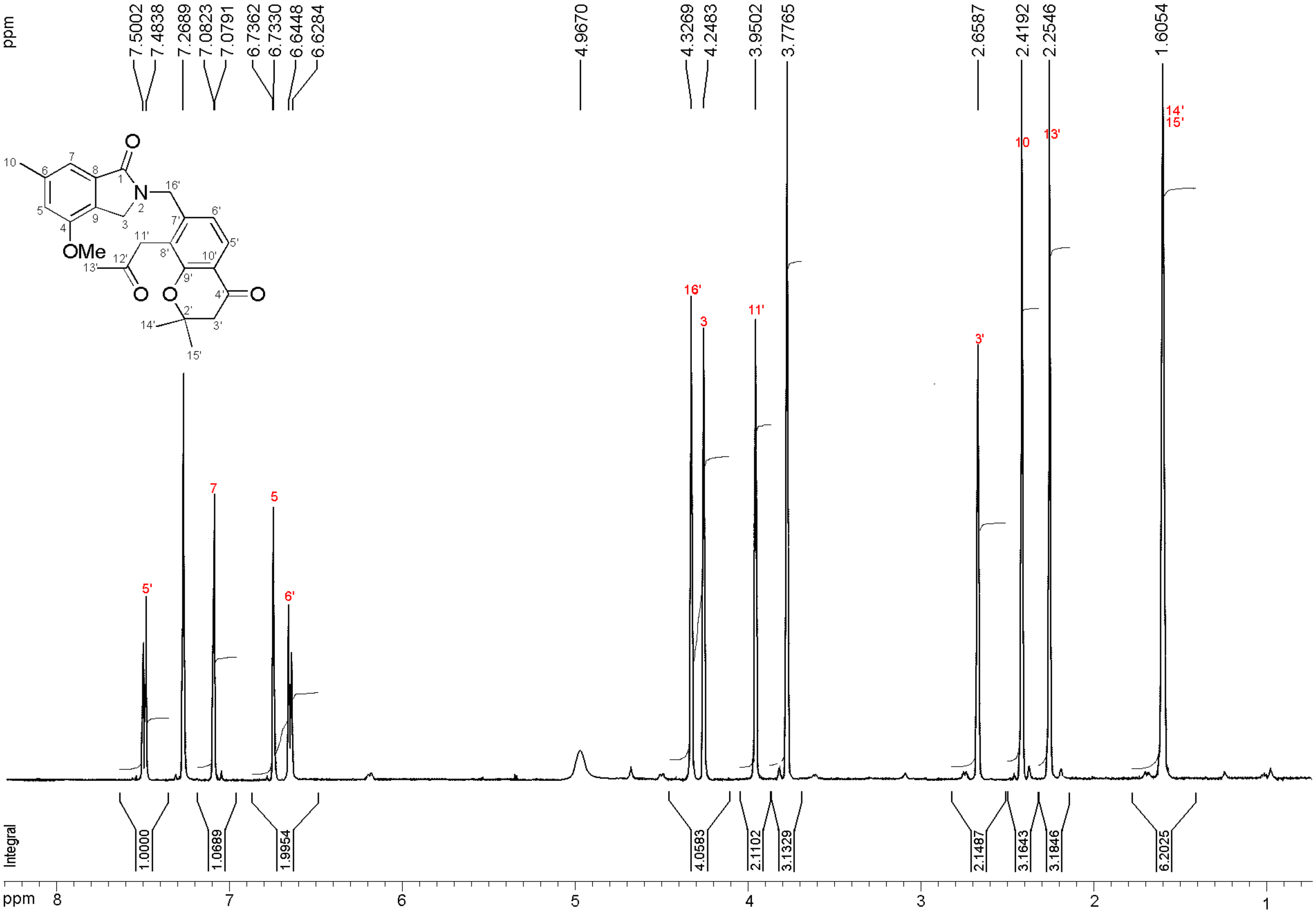

Figure S15. HSQC NMR spectrum of alataindolein D (**5**)

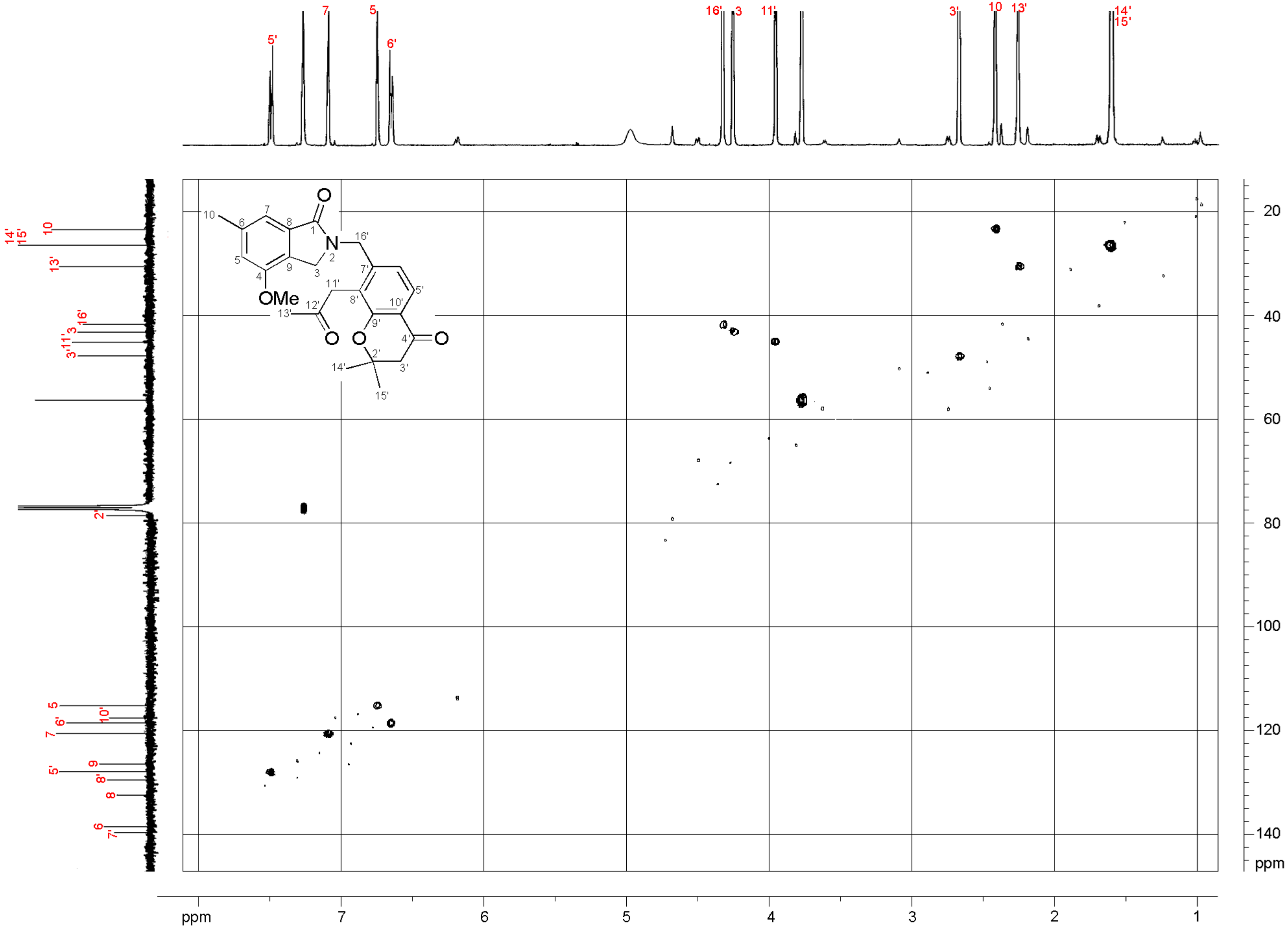

Figure S12. HMBC NMR spectrum of alataindolein D (**5**)

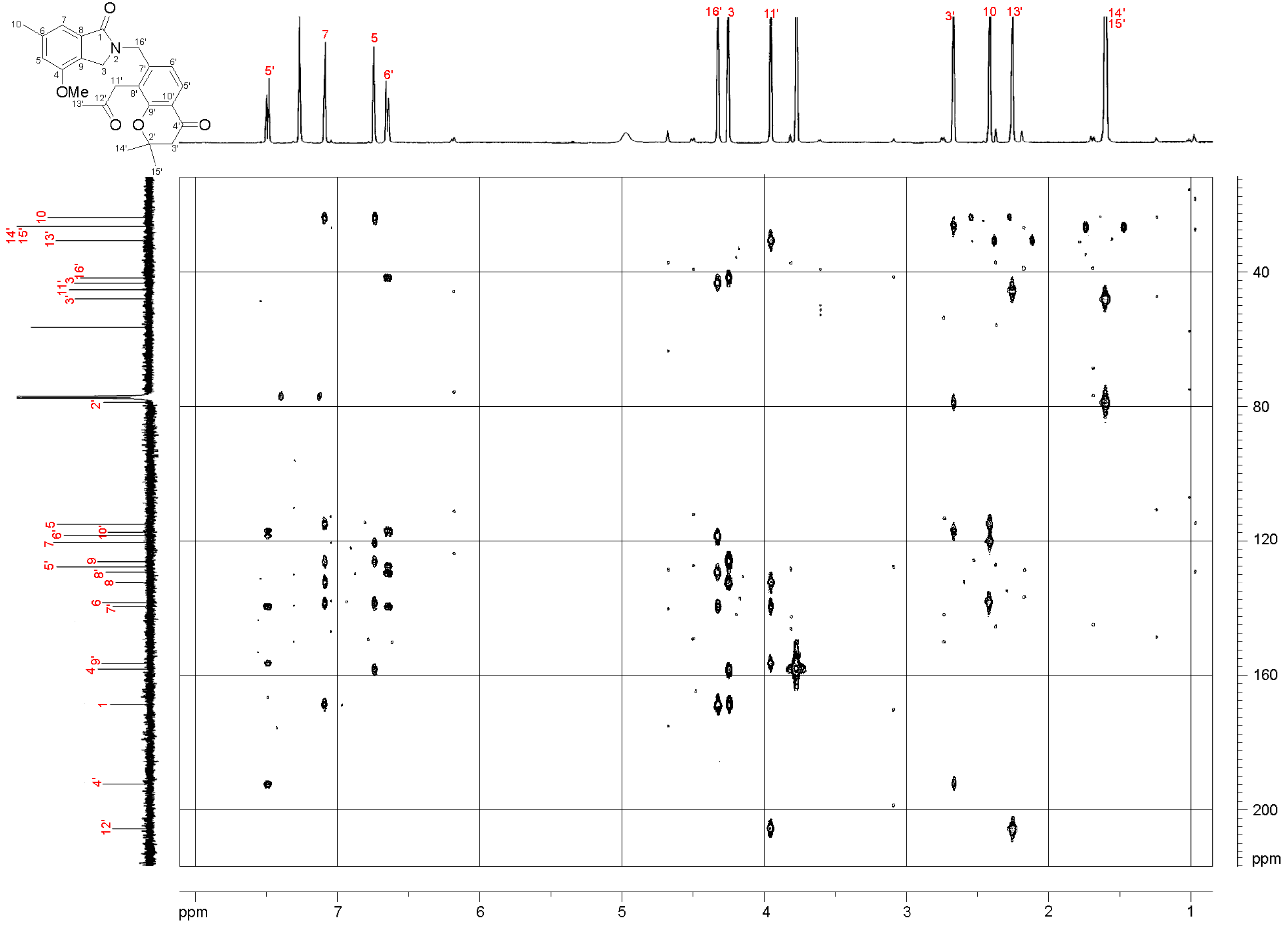

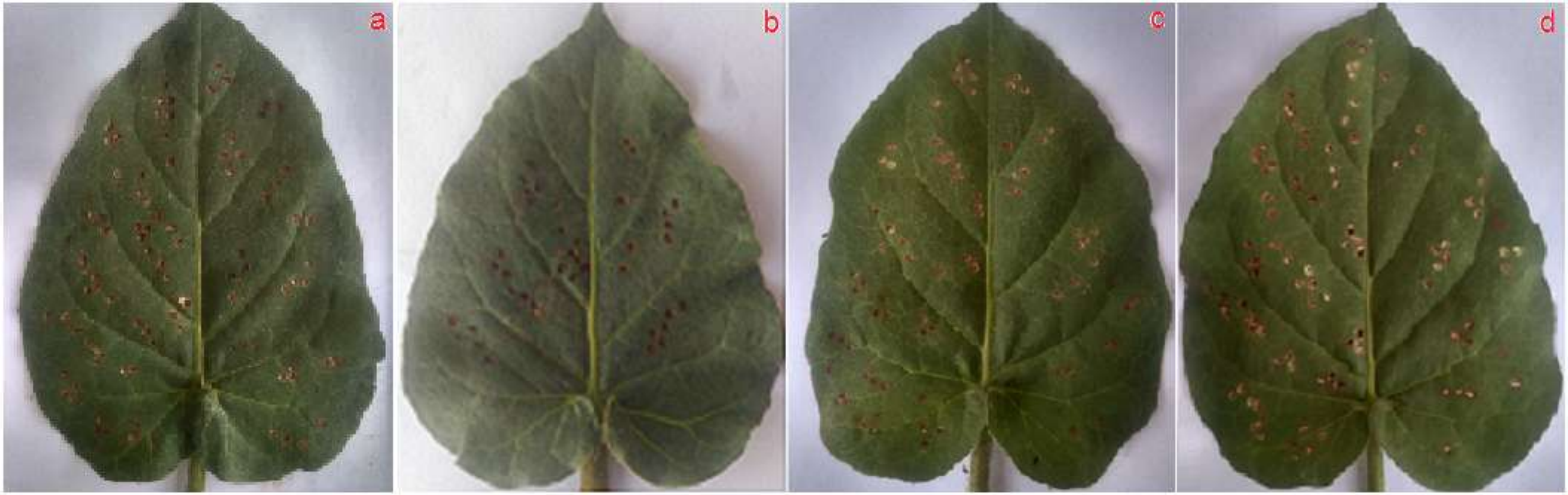

FigFigure S17. The antiviral inhibition rates tested by half-leaf method for compounds **2**–**4**
[**a.** (ningnanmycin); **b.** (compound **2**); **c.** (compound **3**); **d.** (compound **4**)]

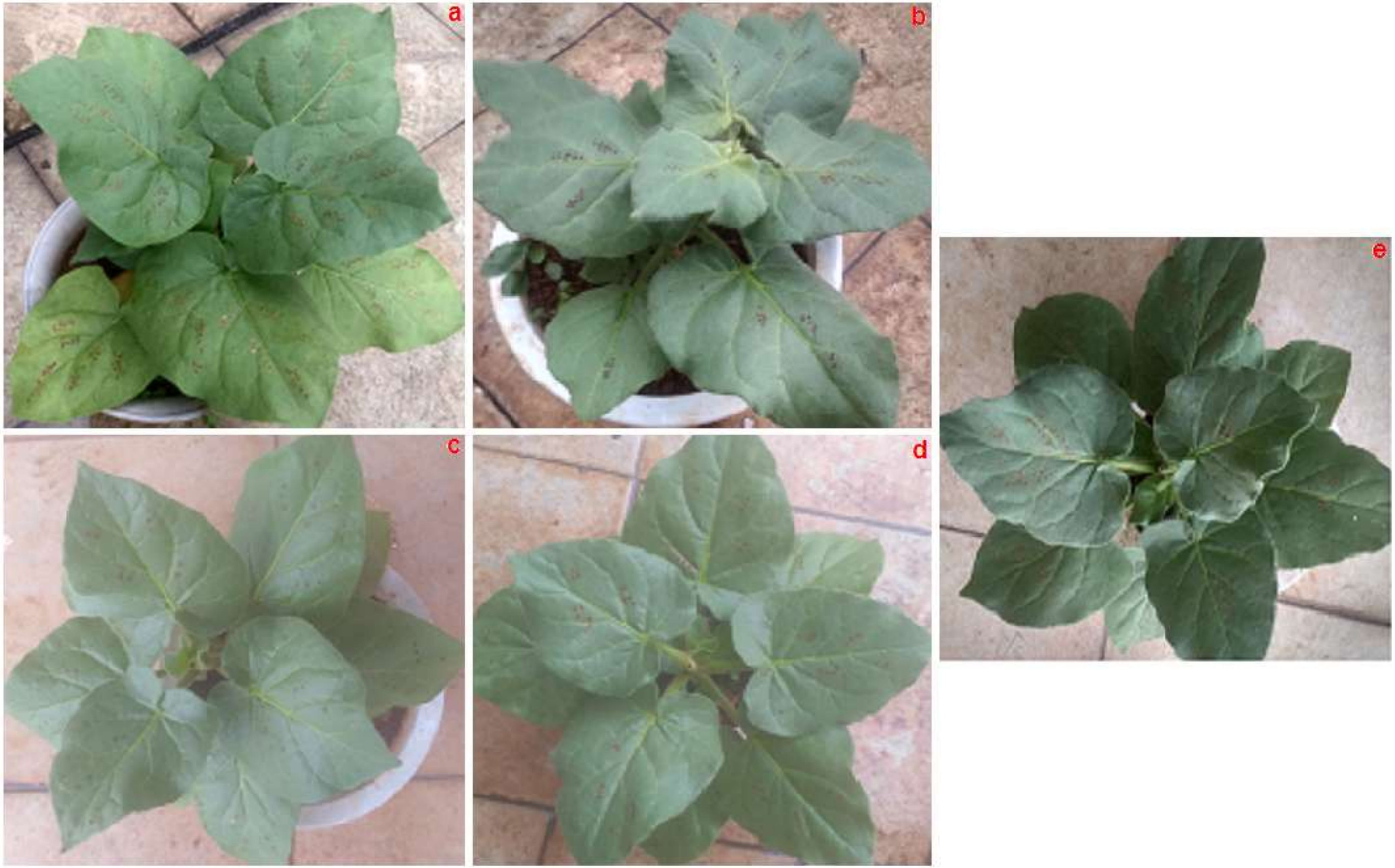

Figure S18. The protective effects of compounds **2**–**4** on TMV

[**a.** control (pretreating the tobacco plant with DMSO soluction); **b.** pretreating the tobacco plant with **2**; **c.** pretreating the tobacco plant with **3**; **d.** pretreating the tobacco plant with **4**; **e.** pretreating the tobacco plant with ningnanmycin (positive control)]
